# Supplementary material for: Substrate specificity of the TRAMP nuclear surveillance complexes
Source: Nat Commun. 2020 Jun 19;11:3122. doi: 10.1038/s41467-020-16965-4 (PMC7305330; doi:10.1038/s41467-020-16965-4)
Supplement: Supplementary file 1 — Supplementary Information [file 41467_2020_16965_MOESM1_ESM.pdf]

Supplementary Information

Substrate Specificity of the TRAMP Nuclear Surveillance Complexes

Clémentine Delan-Forino, Christos Spanos, Juri Rappsilber, David Tollervey

SUPPLEMENTARY FIGURES:

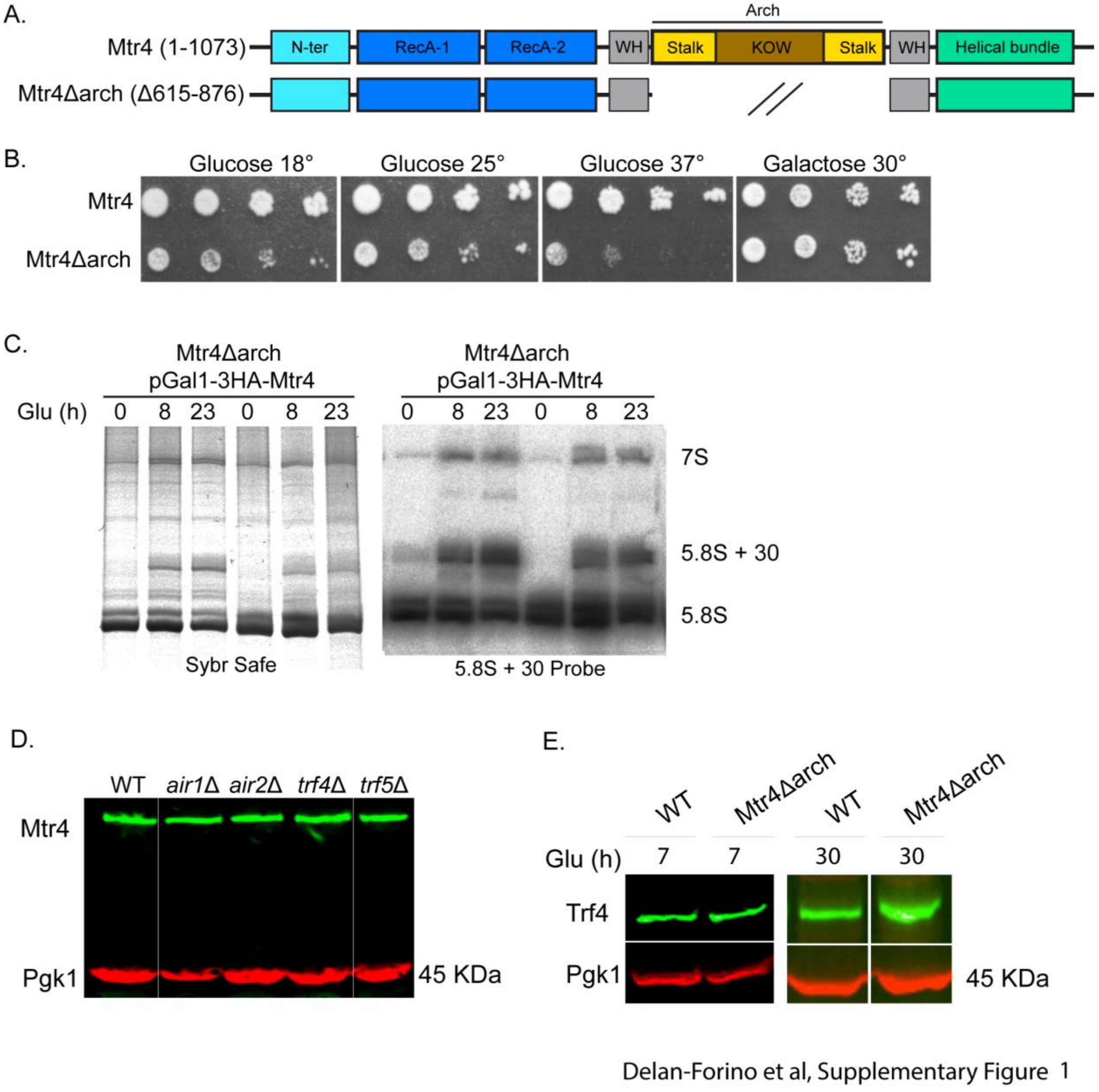

Supplementary Figure 1: Mtr4Δarch construct and phenotype.

A. Main domains of Mtr4 and Mtr4Δarch proteins.

B. Serial dilution (1:5) growth complementation tests of strains expressing Mtr4 and Mtr4Δarch, on galactose medium for expression of wild-type Mtr4, or on glucose to deplete Mtr4. This experiment was performed independently three times and showed similar results.

C. Analysis of 5.8S +30 pre-rRNA, a characterized target requiring the Mtr4 Arch domain for processing, in strains expressing Mtr4Δarch during depletion of wild-type Mtr4. Left panel; SybrSafe stained,10% polyacrylamide, denaturing gel. Right panel; northern blot. Northern blot analysis was repeated twice and showed the same results.

D-E. Western blot analysis assessing abundance of (D) Mtr4 in wild-type strain and strains deleted for TRAMP component and (E) Trf4 in strains expressing wild-type Mtr4 or Mtr4∆arch depleted of wild-type Mtr4 for 7 or 30 h. The same strains were used for CRAC analyses. Western blot analysis was repeated twice and showed the same results.

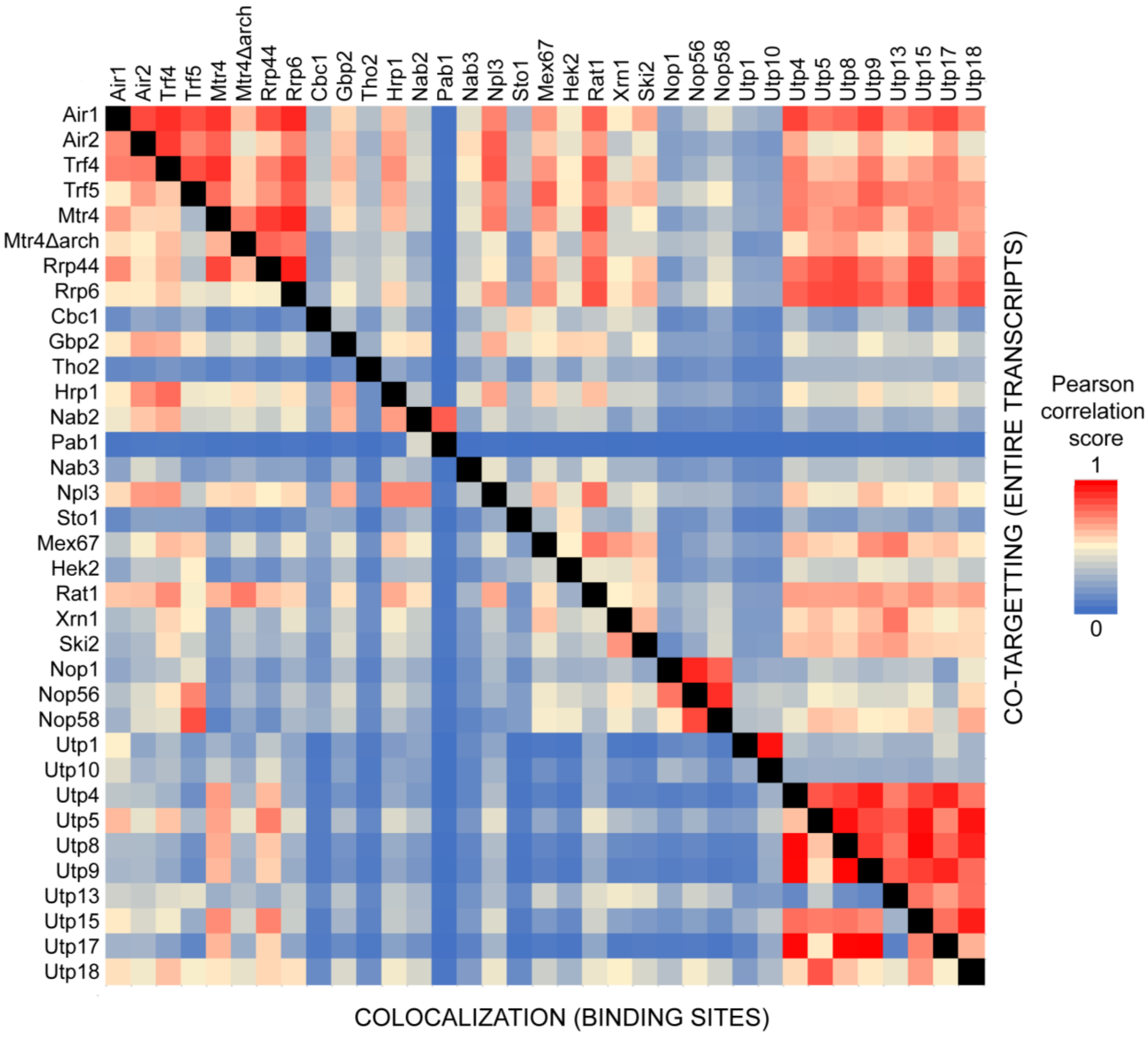

Delan-Forino et al, Supplementary Figure 2

Supplementary Figure 2: Correlations between protein interaction sites over all genes
Correlation matrix for protein binding in CRAC analyses, showing correlations between binding sites. NOTE: Above the diagonal, the matrix shows the extent to which the factors target the same RNA species as “Co-targeting of RNAs”. Below the diagonal, the matrix shows the extent to which the factors target the closely positioned RNA sites (≥50 nt) as “Colocalization of sites”. See Supplementary Data 3 for values for individual CRAC dataset. As Figure 6 but including all annotated genes.

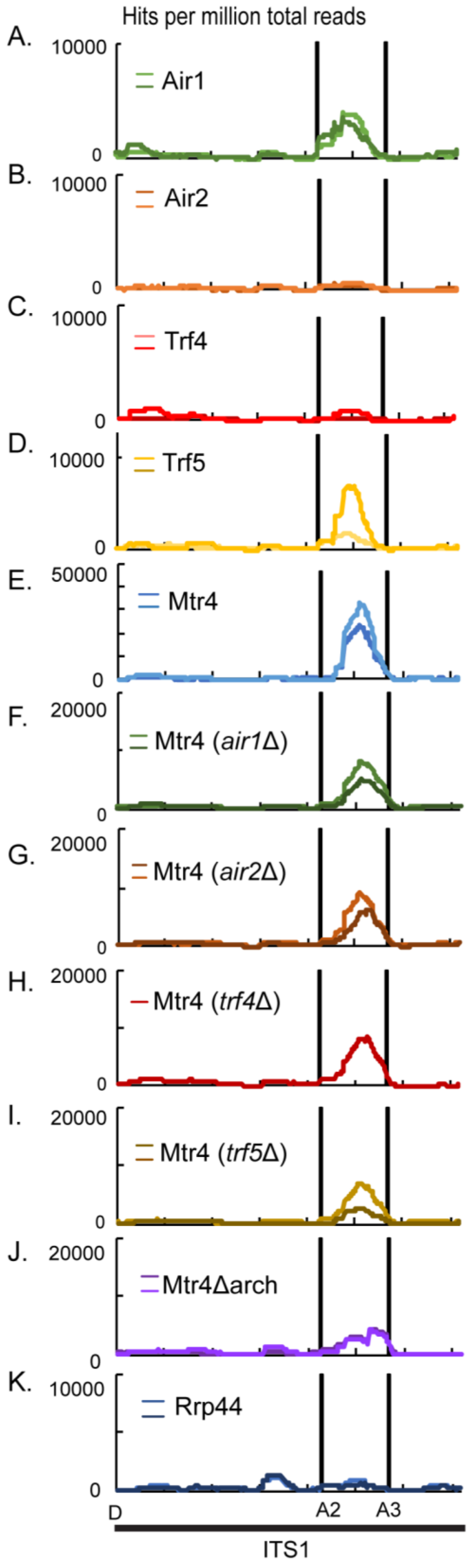

Delan-Forino et al , Supplementary Figure 3

Supplementary Figure 3: TRAMP interaction sites on the pre-rRNA ITS1 region.

A-L. Distribution of total reads across the ITS1 region of the pre-rRNA, normalized to millions of mapped reads and recovered with the indicated TRAMP components (A-E), Mtr4 in strains lacking the indicated TRAMP components (F-I), Mtr4 lacking the Arch domain (J), Rrp44 (K), Rrp44 in strain expressing Mtr4 lacking the Arch domain (L). Scale is linear.

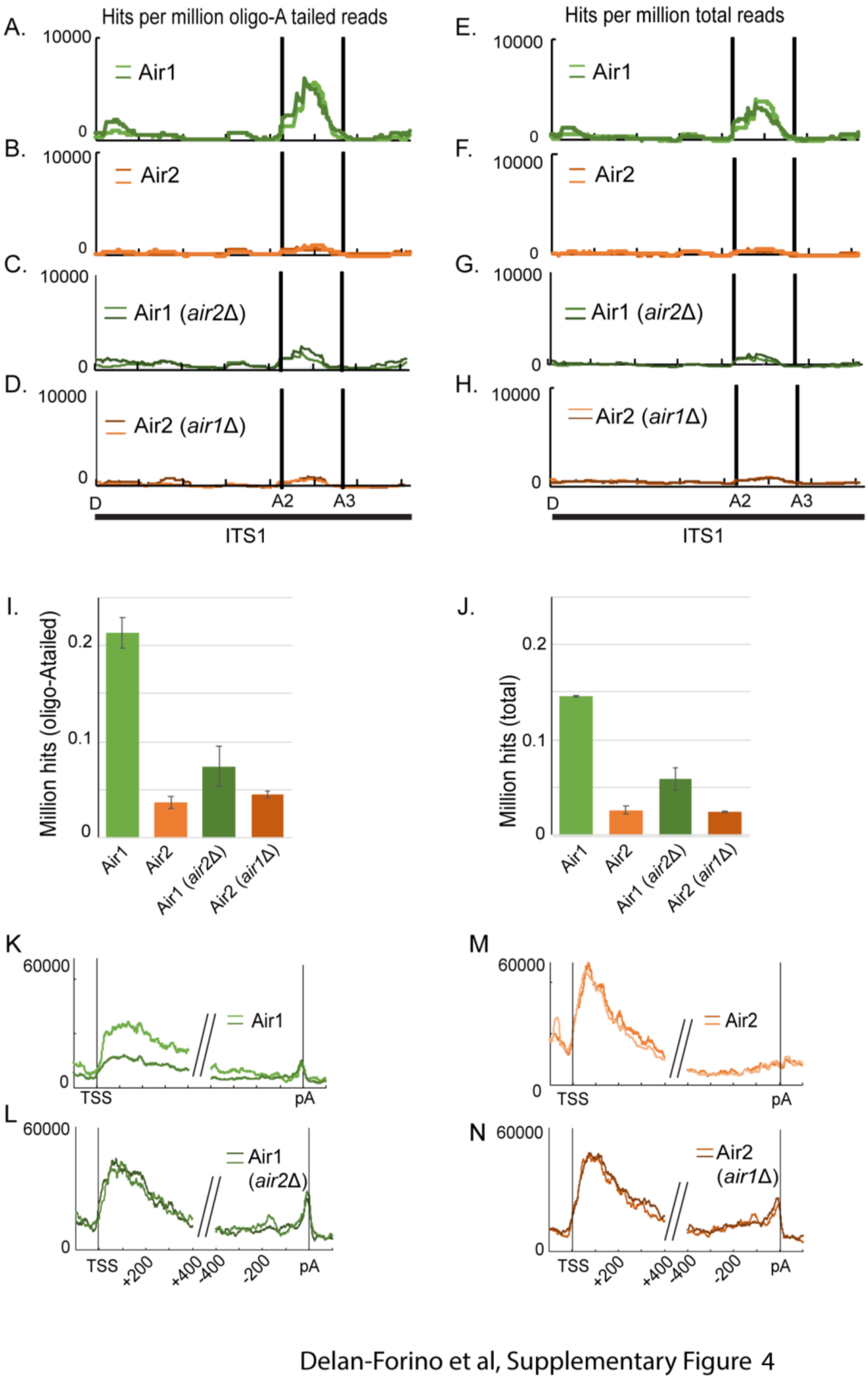

Supplementary Figure 4: TRAMP interaction sites on the pre-rRNA ITS1 region.

A-H. Distribution of reads including non-encoded, oligo(A) tails (A-D) or total reads (E-H) across the ITS1 region of the pre-rRNA, normalized to millions of mapped reads and recovered with Air1 (A, E), Air2 (B, F), Air1 in an *air2*Δ strain (C-G), Air2 in an *air1*Δ strain (D-H). Scale is linear. A diagram of the pre-rRNA region is shown. Two independent replicates are shown in panels as light and dark colored lines.

I-J. Hits per millions oligo(A) tailed reads (I) and total reads (J) encompassing the A2-A3 region of ITS1, extended by 10nt on each side, were summed and are exhibited as a bar diagram. Two individual replicates were averaged, with standard deviation shown as error bars.

K-N. Distribution of individual components indicated, across all mRNAs longer than 500 nt. Each panel shows the hit density, normalized to millions of reads mapped to mRNAs. The two lines in each panel represent results from independent CRAC experiments. Reads were aligned with transcription start sites (TSS) and polyadenylation sites (pA).

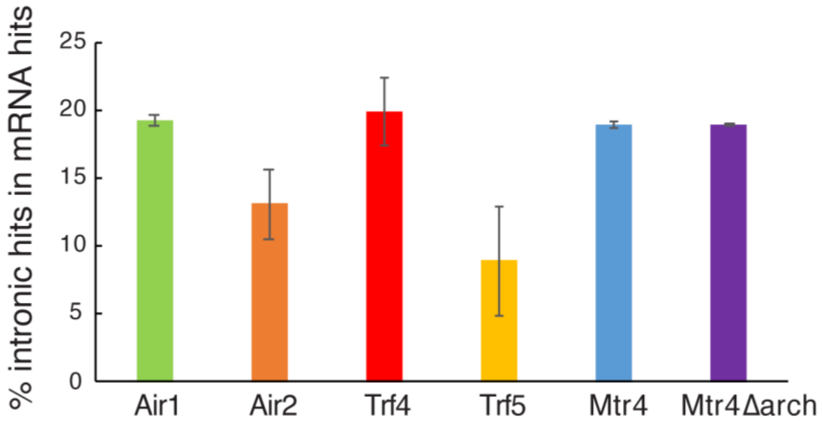

Delan-Forino et al, Supplementary Figure 5

Supplementary Figure 5 Recovery of pre-mRNA intronic sequences in CRAC analyses
The analysis was performed using read per kilobase (sum of intron hits/ sum of all mRNA hits) on collapsed CRAC dataset in which low complexity reads were filtered out. Two individual replicates were averaged, with standard deviation shown as error bars.

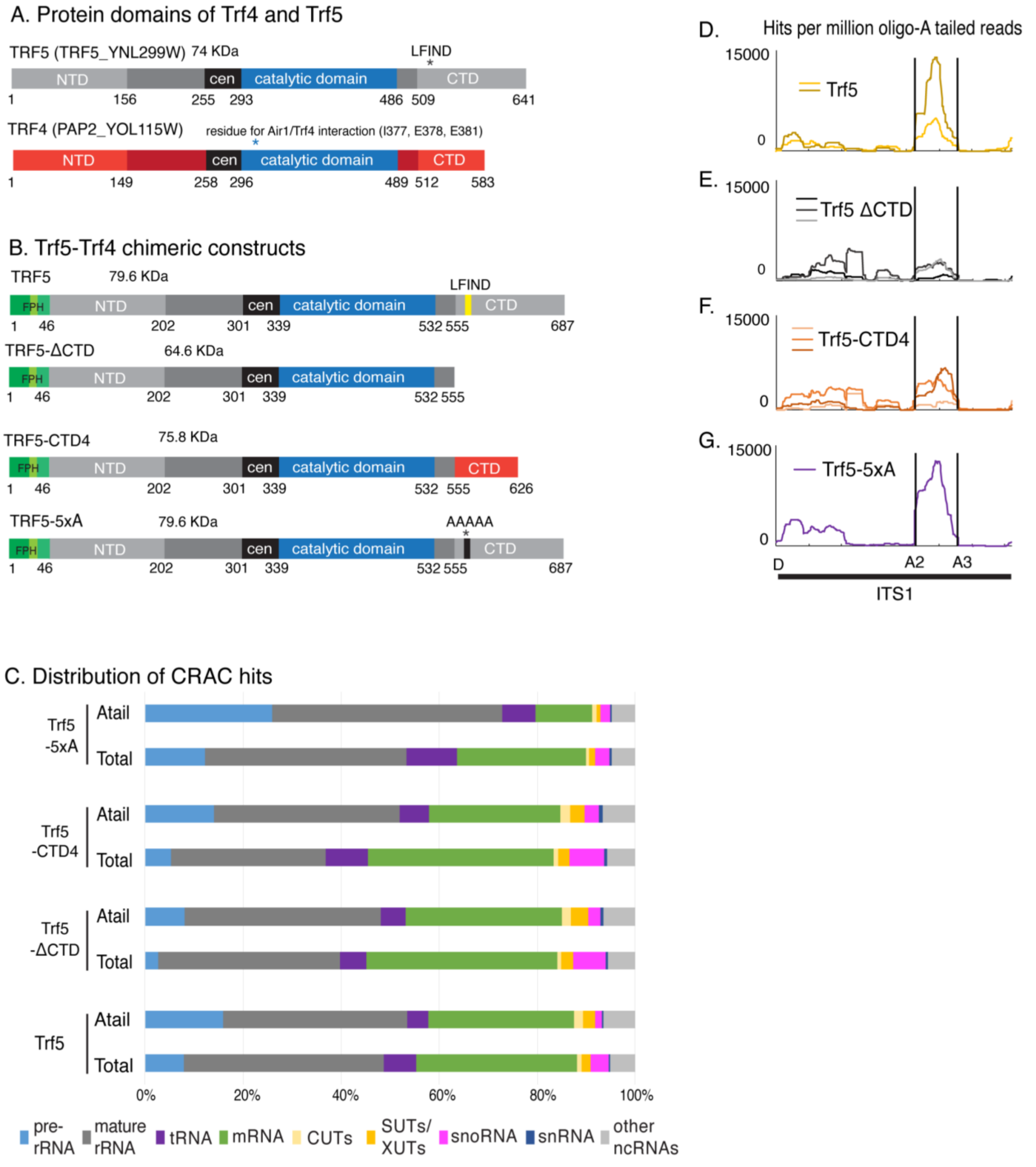

Supplementary Figure 6: Effects of altered Trf5 structure on ITS1 binding

A-B. Schematic representation of major protein domains of Trf4 and Trf5 (A) and Trf5 chimeric constructs used in CRAC analysis (B). NTD: N-terminal domain; CTD: C-terminal domain; cen: central domain; FPH: 3xFlag - Pre-scission protease site - Histidine x 6 Tag.

C. Distribution of all reads and A-tailed reads mapped to different RNA substrate classes recovered in CRAC datasets. At least two biological replicates were averaged for each protein.
D. Distribution of A-tailed reads across the ITS1 region of the pre-rRNA, normalized to millions of mapped reads and recovered with the different Trf5 constructs. The lines in each panel represent results from independent CRAC experiments.

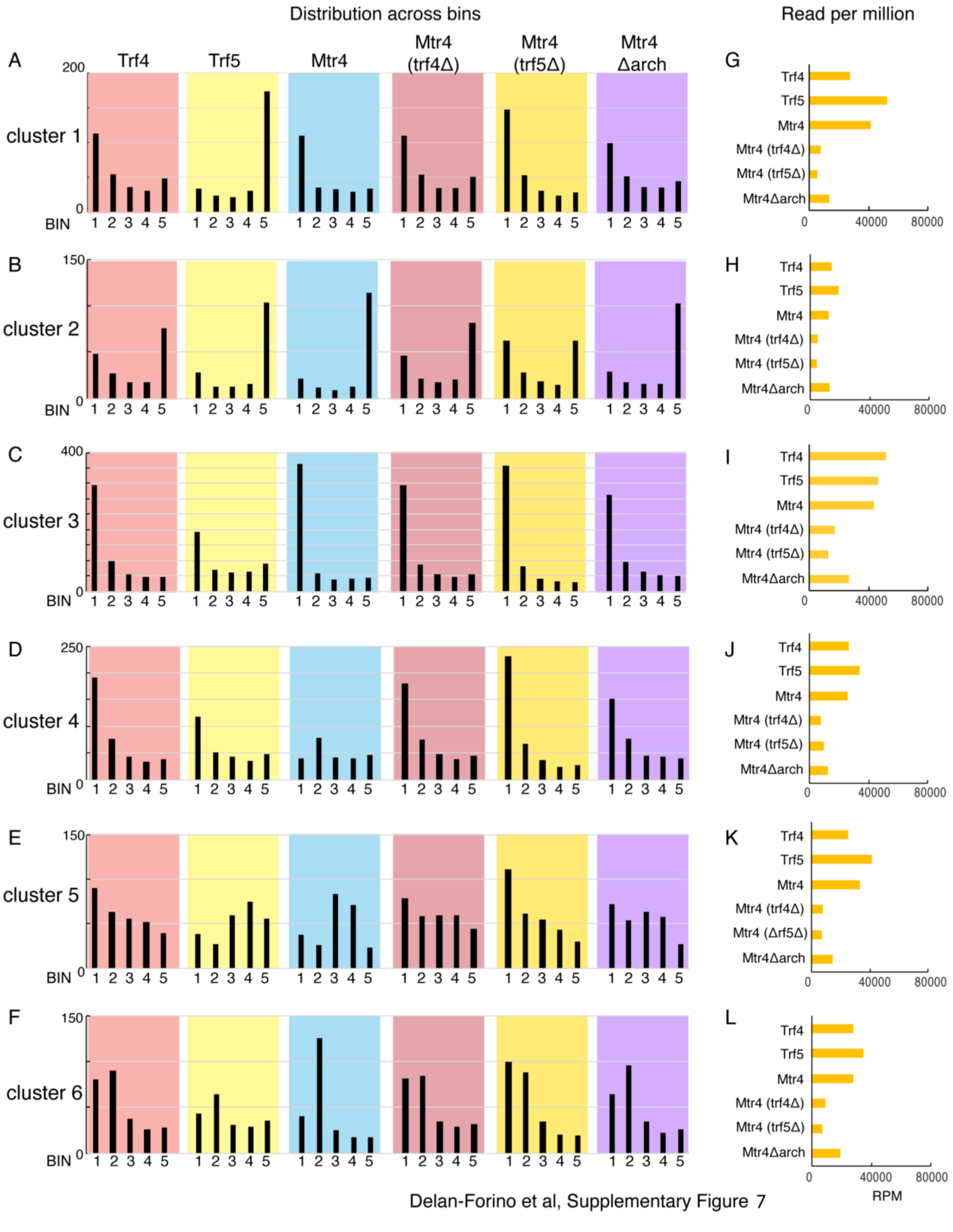

Supplementary Figure 7: Quantitation of total reads in cluster analysis

A-F: Distribution of total hits across mRNAs in each cluster defined in Figure 5. Each transcript was divided in 5 bins of equal length from TSS to pA site. Binding across each bin was calculated as a fraction of total binding across individual gene (set to 1). For each transcript, the average between two biological replicates was used. In each cluster, the values of all bins were added to obtain a binding profile.
G-L: Numbers of reads (RPM) for the indicated factor in each cluster.

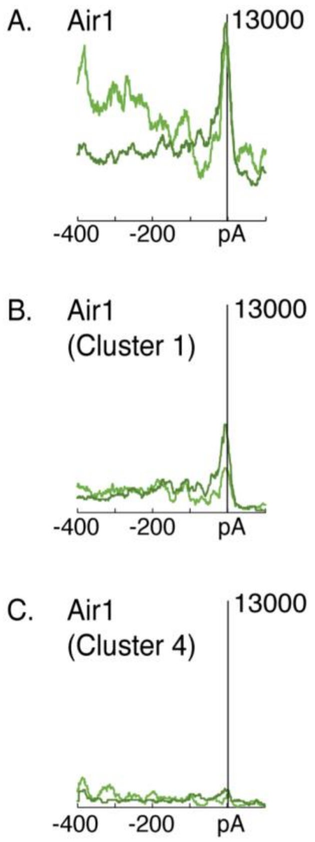

Delan-Forino et al, Supplementary Figure 8

Supplementary Figure 8: Metagene analyses of Air1 around pA sites

A-C. Comparison of Air1 distribution around the pA sites of all mRNAs (A) or in cluster 1 (B) or cluster 4 (C), defined in Figure 5. Each panel shows the hit density, normalized to millions of reads mapped to mRNAs. The two lines in each panel represent results from independent CRAC experiments. Reads were aligned with polyadenylation sites (pA).

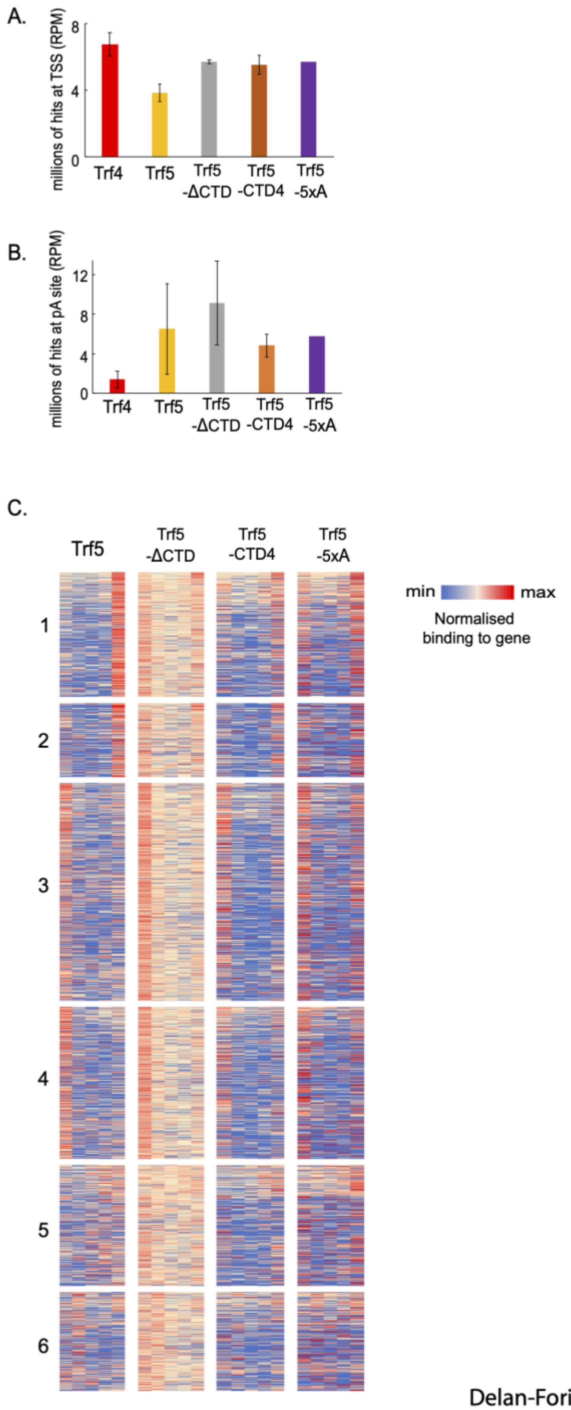

Delan-Forino et al, Supplementary Figure 9

Supplementary Figure 9: Effects of altered Trf5 structure on mRNA binding

A-B. Hits per millions oligo(A) tailed reads encompassing the TSS region (TSS +150 nt) (A) or poly(A) site (pA -150 nt) (B) were summed and exhibited as a bar graph. Two individual replicates were averaged, with standard deviation shown by error bars (except Trf5-5xA for which only one experiment was performed).

C. HEAT map showing binding across individual mRNAs from clusters defined in Figure 5. Each transcript was divided into 5 bins of equal length from TSS to pA site. Binding across each bin was calculated as a fraction of total binding across individual gene (set to 1). Numbers of reads were averaged between two biological replicates. The data are displayed as heat maps. Colors reflect relative binding between different bins of the same mRNA, so the lighter color for the Trf5-ΔCTD panel indicates a more even distribution.

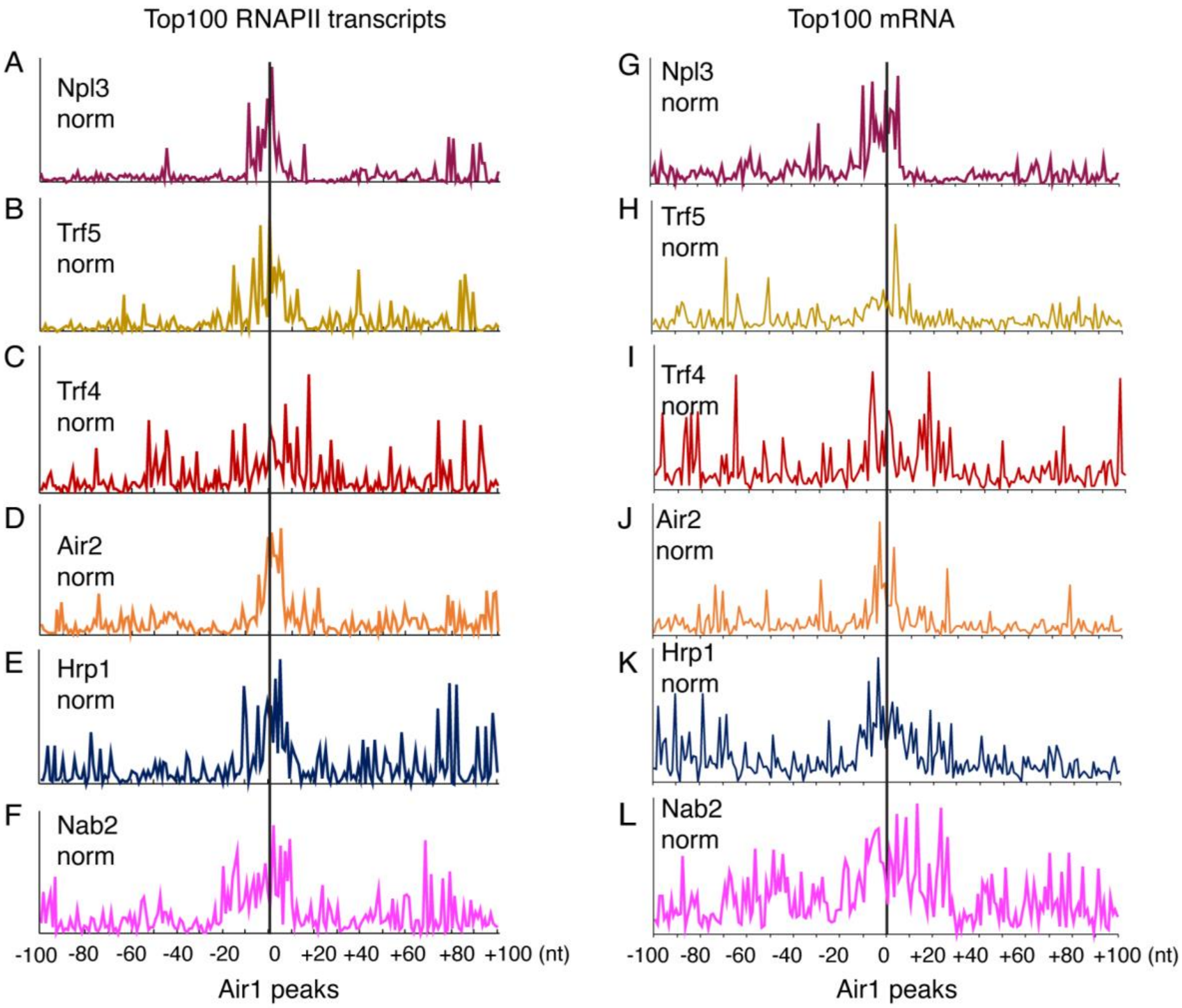

Delan-Forino et al , Supplementary Figure 10

Supplementary Figure 10: Correlations between protein interaction sites at the nucleotide level

As Figures 6B-6M, except the distribution of binding across each gene was calculated as a fraction of total binding across the gene (set to 1).

A-L: Binding of the indicated proteins relative to Air1. CRAC peaks for Air1 across the genome were selected and used as a reference to align peaks for other proteins. Data are shown for the combined top 100 RNAPII transcripts bound by Npl3 and Air1 (A-F, 127 genes in total) or top 100 mRNAs (G-L, 147 genes in total). Two independent replicates were combined in each panel.

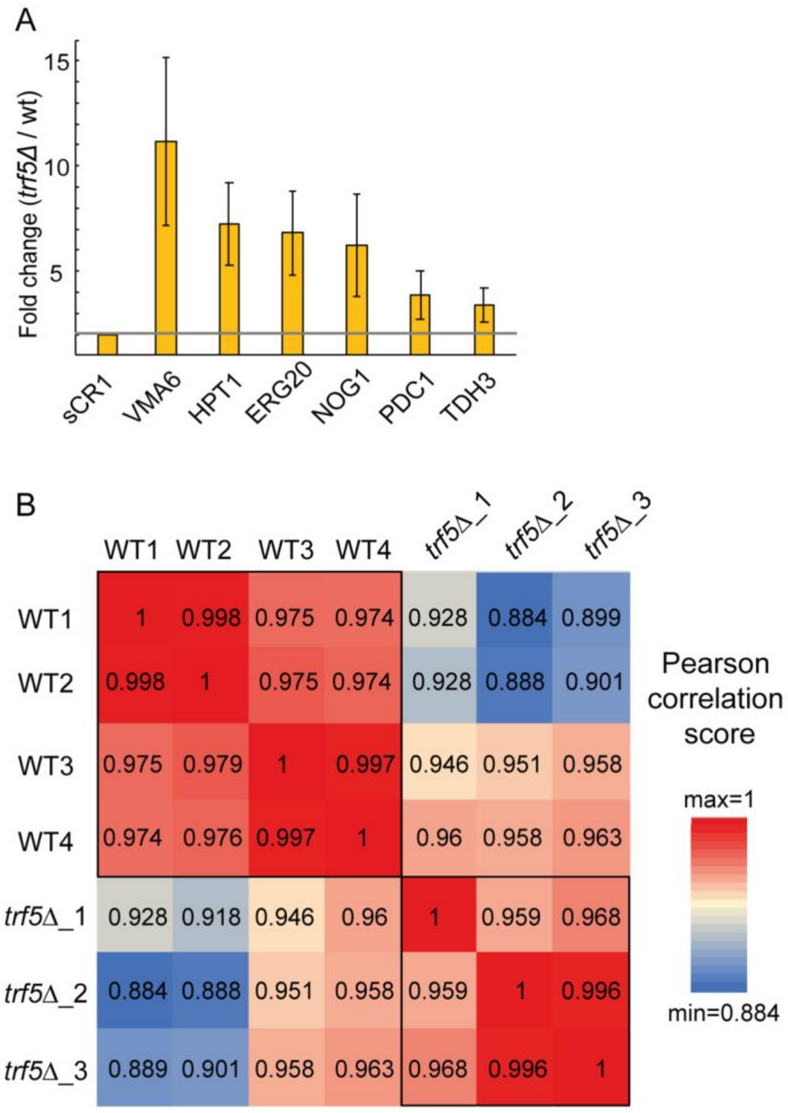

Delan-Forino et al, Supplementary Figure 11

Supplementary Figure 11: Altered mRNA abundance in *trf5*∆ and correlations of RNAseq samples over mRNA genes

A. Gene expression fold change between wild-type and *trf5*∆ on selected genes. Technical triplicates for each independent biological replicates (2 for wild-type and 3 for *trf5*∆) were submitted to Real-time PCR amplification. Primer efficiencies were calculated and cycle threshold (Ct) values were averaged between triplicates for each RNA sample and standard deviation shown as error bars. Gene expression fold change were determined as previously reported [1]. For qPCR raw data, primer efficiencies, and fold change calculation on wild-type and *trf5*∆ strains, see Supplementary Data 7.

B: Correlation matrix of mRNAs recovered between RNAseq replicates (4 wild-type (WT) and 3 *trf5*∆ samples) used for Figure 7.

SUPPLEMENTARY REFERENCE

1 Pfaffl, M. W. A new mathematical model for relative quantification in real-time RT-PCR. *Nucleic Acids Res.* **29**, e45-e45, doi:10.1093/nar/29.9.e45 (2001).
