# Supplementary material for: Substrate specificity of the TRAMP nuclear surveillance complexes
Source: Nat Commun. 2020 Jun 19;11:3122. doi: 10.1038/s41467-020-16965-4 (PMC7305330; doi:10.1038/s41467-020-16965-4)
Supplement: Supplementary file 3 — Reporting Summary [file 41467_2020_16965_MOESM3_ESM.pdf]

nature research

Corresponding author(s): David Tollervey

Last updated by author(s): Dec 19, 2019

Reporting Summary

Nature Research wishes to improve the reproducibility of the work that we publish. This form provides structure for consistency and transparency in reporting. For further information on Nature Research policies, see Authors & Referees and the Editorial Policy Checklist.

Statistics

For all statistical analyses, confirm that the following items are present in the figure legend, table legend, main text, or Methods section.

n/a	Confirmed	
☐	☒	The exact sample size (*n*) for each experimental group/condition, given as a discrete number and unit of measurement
☐	☒	A statement on whether measurements were taken from distinct samples or whether the same sample was measured repeatedly
☐	☒	The statistical test(s) used AND whether they are one- or two-sided<br>*Only common tests should be described solely by name; describe more complex techniques in the Methods section.*
☒	☐	A description of all covariates tested
☒	☐	A description of any assumptions or corrections, such as tests of normality and adjustment for multiple comparisons
☐	☒	A full description of the statistical parameters including central tendency (e.g. means) or other basic estimates (e.g. regression coefficient) AND variation (e.g. standard deviation) or associated estimates of uncertainty (e.g. confidence intervals)
☒	☐	For null hypothesis testing, the test statistic (e.g. *F*, *t*, *r*) with confidence intervals, effect sizes, degrees of freedom and *P* value noted<br>*Give P values as exact values whenever suitable.*
☒	☐	For Bayesian analysis, information on the choice of priors and Markov chain Monte Carlo settings
☒	☐	For hierarchical and complex designs, identification of the appropriate level for tests and full reporting of outcomes
☐	☒	Estimates of effect sizes (e.g. Cohen's *d*, Pearson's *r*), indicating how they were calculated

Our web collection on statistics for biologists contains articles on many of the points above.

Software and code

Policy information about availability of computer code

Data collection	Software used for data collection was the proprietary software that belongs to the machines described in the Methods section (Illumina HiSeq (Edinburgh Genomics) or Illumina Miniseq (our laboratory); Q Exactive Mass Spectrometer (Thermo Fisher Scientific) coupled on-line, to an Ultimate 3000 RSLCnano Systems (Dionex, Thermo Fisher Scientific))
Data analysis	Code is described and accurately cited. Most of the code used in this work is available online. Custom code can be made available upon request through the corresponding author. Software used were Flexbar 3.4.0 57 with parameters -x 1 –ao 4 -g; Novoalign (Novocraft, V2.07) with genome annotation from Ensembl (EF4.74); pyCRAC (0.5.3); STAR; Cluster3.0 (C Clustering Library version 1.52a); MaxQuant (version 1.6.1. 0); MView 1.63

For manuscripts utilizing custom algorithms or software that are central to the research but not yet described in published literature, software must be made available to editors/reviewers. We strongly encourage code deposition in a community repository (e.g. GitHub). See the Nature Research guidelines for submitting code & software for further information.

Data

Policy information about availability of data

All manuscripts must include a data availability statement. This statement should provide the following information, where applicable:

- Accession codes, unique identifiers, or web links for publicly available datasets
- A list of figures that have associated raw data
- A description of any restrictions on data availability

The mass spectrometry proteomics data have been deposited to the ProteomeXchange Consortium via the PRIDE partner repository with the dataset identifier PXD017114.
All sequence data from experiments made for this study and Rat1 datasets from Grannemanet al, EMBO, 2011 are available from NCBI's Gene Expression Omnibus (GEO) under accession number GSE135526. Published data are available under GEO accession number GSE77683 (Mtr4), GSE69696 (Rrp44, Rrp6, Air2), GSE79950 (UTP proteins), GSE70191 (Nab3, Npl3, Sto1), GSE114680 (Nop1, Nop56, Nop58), GSE46742 (Trf4, Cbc1, Gbp2, Tho2, Hrp1, Nab2, Pab1, Mex67, Hek2, Xrn1, Ski2).
We used Saccharomyces cerevisiae genome version (SGD v64) with genome annotation from Ensembl (EF4.74) for analysis.

Associated raw data for:
-figs 1C, 1E are available from supplementary tables S2 and S3 respectively
-fig 2C and S2 are available from supplementary table S4
-fig. 5A and supplementary figure S6 are available from supplementary table S5
-supplementary figure S10A are available from supplementary table S7
-figs 2A, 2B, 3A-0, 4A-P, 6, 7 and supplementary figures S1C-E, S3, S4, S5C-G, S7, S8, S9 are available from source data file

Field-specific reporting

Please select the one below that is the best fit for your research. If you are not sure, read the appropriate sections before making your selection.

☒ Life sciences ☐ Behavioural & social sciences ☐ Ecological, evolutionary & environmental sciences

For a reference copy of the document with all sections, see nature.com/documents/nr-reporting-summary-flat.pdf

Life sciences study design

All studies must disclose on these points even when the disclosure is negative.

Sample size	CRAC analyses were conducted at least in duplicate (Except Mtr4 Trf4delta CRAC and Trf5-5xA).<br>qPCR analyses have been performed on 2 biological replicates for wild-type and three for Trf5delta, each of them analysed in technical triplicates.<br>Mass Spectrometry and Western blot were performed in triplicate or duplicate.<br>Replicates have been highly similar most of the time, therefore we did not consider that adding more repeats was required
Data exclusions	One Trf5 mass spectrometry replicate sample has been excluded due to poor signal to noise ratio. Trf5 mass spectrometry analyses are supporting conclusions made from every other protein pull-down analysis and consistent with published litterature.
Replication	All findings reported were shown to be reproducible (duplicates or triplicates were conducted for CRAC, Mass spectrometry, qPCR, Western blot and Northern blot) unless stated otherwise in the manuscript (in particular for CRAC experiments performed on Trf5 chimeric constructs described in FigS5 and S8)
Randomization	Randomization is not done in this type of yeast study. All results were quantified with data analysis tools and not based on visual observation that could lead to bias.
Blinding	Blinding is not done in this type of yeast study. All results were quantified with data analysis tools and not based on visual observation that could lead to bias.

Reporting for specific materials, systems and methods

We require information from authors about some types of materials, experimental systems and methods used in many studies. Here, indicate whether each material, system or method listed is relevant to your study. If you are not sure if a list item applies to your research, read the appropriate section before selecting a response.

Materials & experimental systems

n/a	Involved in the study	
☐	☒	Antibodies
☒	☐	Eukaryotic cell lines
☒	☐	Palaeontology
☒	☐	Animals and other organisms
☒	☐	Human research participants
☒	☐	Clinical data

Methods

n/a	Involved in the study	
☒	☐	ChIP-seq
☒	☐	Flow cytometry
☒	☐	MRI-based neuroimaging

Antibodies

Antibodies used	rabbit anti-TAP antibody (Thermo Scientific CAB1001 and mouse anti-Pgk1 antibody (Thermo Fisher PA528612) were used as primary antibodies (1:5000), followed by Li-cor IRDye secondary antibodies (dilution 1:10000): anti-mouse 680RD (926-68070) and anti-rabbit 800CW (926-32211) incubation.
Validation	TAP is species-independent as stated on product homepage. Anti-TAP CAB1001 has been tested by the manufacturer in these conditions : Western blot analysis of TAP tagged RPP1B was performed by loading 10 uL of yeast extract and 7 µL of Molecular Weight Protein Ladder per well onto a 4-20% Bis-Tris polyacrylamide gel. Proteins were transferred to a nitrocellulose membrane and stained/destained with Pierce MemCode Blue Kit (Product # 24580). Membranes were probed with Pierce Fast Western Kit (Product # 35050). Proteins were stained with rabbit polyclonal antibodies recognizing the TAP tag (Product # CAB1001). CAB1001, at 0.5 µg/mL, at 1.0 µg/mL, and 0.25 µg/mL were incubated for 30 minutes at room temperature on a rocking platform per Fast Western Kit instructions. Membranes were then washed in kit wash buffer and probed with kit

secondary antibody reagent for 10 minutes diluted at 150 uL per 10 mL kit reagent diluent. Membranes were washed in kit wash buffer and chemiluminescent detection was performed using Pierce Super Signal West Dura (Product # 34075). CAB1001 detects TAP. CAB1001 has been successfully used in Western Blot, IP, and ChIP applications. The CAB1001 immunogen is a KLH conjugated peptide representing the C-terminus of the TAP construct after TEV cleavage. TAP adds about 20 kDa to the size of the protein.

Anti-Pgk1 antibody species reactivity for yeast is confirmed on product homepage. According to manufacturer "Antibody specificity was demonstrated by siRNA mediated knockdown of target protein. HeLa cells were transfected with PGK1 siRNA and loss of signal was observed in Western Blot using Anti-PGK 1 Polyclonal Antibody". This antibody has been used routinely for yeast applications in our laboratory and the size of the band is consistent with the expected size of Pgk1.
