# Supplementary material for: Substrate specificity of the TRAMP nuclear surveillance complexes
Source: Nat Commun. 2020 Jun 19;11:3122. doi: 10.1038/s41467-020-16965-4 (PMC7305330; doi:10.1038/s41467-020-16965-4)
Supplement: Supplementary file 4 — Description of Additional Supplementary Files [file 41467_2020_16965_MOESM4_ESM.pdf]

Description of Additional Supplementary Files

File Name: Supplementary Data 1
Description: Strains used in this study

File Name: Supplementary Data 2
Description: iBAQ (Intensity based absolute quantification) values for proteins recovered by mass spectrometry (LC-MS/MS) analysis. Air1, Air2, Trf4 and Trf5 were used as bait for pull-downs in buffer containing 1M NaCl. IBAQ values in the mock precipitated sample are shown as the negative control.

File Name: Supplementary Data 3
Description: riBAQ (relative molar abundances for each protein) values for proteins recovered by mass spectrometry (LC-MS/MS) analysis. The first sheet shows rIBAQ ratios, determined by dividing the iBAQ value for each protein by the sum of all noncontaminant iBAQ values. The second sheet shows enrichment, calculated as the riBAQ ratio between TRAMP protein pull-down and the mock sample. All proteins showing a ratio less than 2 were considered to be not significantly enriched.

File Name: Supplementary Data 4
Description: Correlation matrix showing Pearson correlation between binding sites recovered with different factors. The first sheet shows correlations calculated from hits across individual transcript (the extent to which the factors target the same RNA species as "Co-targeting of RNAs"). The second sheet shows correlations calculated from hits across 50 nt windows encompassing the whole genome (the extent to which the factors target closely positioned RNA sites (≥50 nt) as "Colocalization of sites"). Values for individual CRAC datasets are shown.

File Name: Supplementary Data 5
Description: Heat maps for distribution of TRAMP components on each mRNA. Heat map showing binding across individual mRNAs of different factors. The top 1,000 protein-coding genes recovered in CRAC with each TRAMP component were selected and combined (2,005 mRNAs in total). Each transcript was divided into 5 bins of equal length from TSS to pA site. Binding across each bin was calculated as a fraction of total binding across individual genes (set to 1). Numbers of reads were averaged between two biological replicates. Only the coverage of Air1, Air2, Trf4, Trf5 and Mtr4 along each transcript was used for Euclidian distance-based clustering. Other data sets shown were not included in the clustering analysis but are sorted according to the clustered list. The data are displayed as heat maps. The RPM for each transcript recovered by each protein was calculated and represented as independent heat maps. Displayed in Figure 5.

File Name: Supplementary Data 6
Description: Correlation matrix showing Pearson correlation between binding sites recovered with different factors across RNA polymerase II transcripts. The first sheet shows correlations calculated from hits across individual RNAPII transcript (the extent to which the factors target the same RNA species as "Co-targeting of RNAs"). The second sheet shows correlations calculated from hits across 50 nt windows encompassing the parts of genome

coding for RNAPII transcripts (the extent to which the factors target the closely positioned RNA sites (≥50 nt) as “Colocalization of sites”). Values for individual CRAC datasets are shown.

File Name: Supplementary Data 7
Description: Raw data, Primer efficiencies, and fold change calculation for Real-time PCR amplification analysis on wild-type and trf5Δ strains. Technical triplicates for each Independent biological replicates (2 for wild-type and 3 for trf5Δ) were submitted to qPCR. Primer efficiencies were calculated and cycle threshold (Ct) values were averaged between triplicates for each RNA sample. Gene expression fold change were determined as previously reported[1] .
